# Self-referenced Digital Spectral Chromatic Local Surface Plasmon Resonance in Ultrasensitive Severe Sepsis Interleukin-6 Detection

**DOI:** 10.1021/acssensors.4c03067

**Published:** 2025-02-05

**Authors:** Ting-Wei Chang, Ting-Hao Chuang, Sheng-Hann Wang, Wing Kiu Yeung, Pei-Kuen Wei

**Affiliations:** † Research Center for Applied Sciences, Academia Sinica, Taipei 115201, Taiwan; ‡ Nano Science and Technology Program, Taiwan International Graduate Program, Academia Sinica, Taipei 11529, Taiwan; § Department of Engineering and System Science, National Tsing Hua University, Hsinchu 300, Taiwan; ∥ Department of Materials and Mineral Resources Engineering, National Taipei University of Technology, Taipei 10608, Taiwan

**Keywords:** local surface plasmon resonance (LSPR), gold nanoparticles, spectral chromatic images, interleukin-6 (IL-6), digital sensing, RGB color image

## Abstract

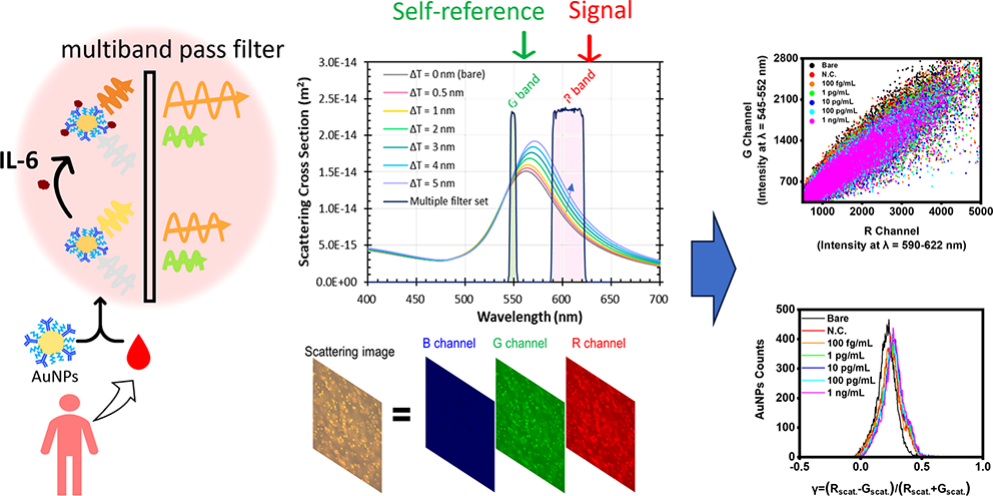

Clinical monitoring of cytokines, such as interleukin-6 (IL-6), enables a timely diagnosis and can significantly improve patient prognosis. In this study, we developed a rapid, label-free, ultrasensitive, and low matrix-effect method called chromatic digital nanoplasmon-metry (cDiNM) to detect IL-6 in human blood plasma. Utilizing a multiple filter configuration, two nonadjacent specific transmission wavelength bands are extracted. One is centered within the full-width-at-half-maximum (fwhm) range where the local surface plasmon resonance (LSPR) response of the 80 nm gold nanoparticles (AuNPs) is strongest, while the other band is narrowed and blue-shifted from the peak to a region with minor intensity change. Scattering images of AuNPs passing through these two bands are then captured simultaneously and independently via the red and green channels of a color scientific complementary metal–oxide–semiconductor (sCMOS) camera. This configuration allows every AuNPs’ spectral chromatic image contrast to be a self-referenced subtractive analysis LSPR and facilitates evaluation of their changes induced by the IL-6 binding across numerous individual AuNPs. This method achieves IL-6 detection in blood plasma within 45 min, requiring only 0.5 mL of a 10-fold diluted, label-free sample, with a limit of detection and quantification (LOD and LOQ) of less than 19.2 and 87.8 fg/mL, respectively, and a recovery rate of 96%. In summary, cDiNM provides rapid and accurate IL-6 monitoring with promising potential for clinical application in sepsis patient care.

Human interleukin-6 (IL-6) is a 21–26 kDa glycoprotein composed of 184 amino acids. During tissue infection and inflammation, IL-6 is secreted by leukocytes, acting as a cytokine for mediating immune responses. It can regulate the production of immunoglobulin by mediating the differentiation of activated B cells into immunoglobulin-producing plasma cells.^1^ Yet, the dysregulation of IL-6 would also be harmful, such as rheumatoid arthritis, one kind of chronic inflammation in which excessive IL-6 was detected in synovial fluids from the joints of patients.^2^ Apart from immune responses, IL-6 is reported to have an influence on energy control, for example, lipids, glucose, and protein metabolism.^3^

Among these physiological effects affected by IL-6, sepsis is a dysregulated systemic immune response to infection. Particularly, clinical deterioration from sepsis to septic shock might be rapid and lethal. In healthy human blood, the relevant plasma IL-6 levels are less than 10 pg/mL, while in sepsis patients, they can exceed 1600 pg/mL.^4,5^ Therefore, developing quick and high-precision equipment for monitoring IL-6 can provide prompt clinical intervention for patients.^6^

Current common methods in clinical settings for IL-6 monitoring in blood are enzyme-linked immunosorbent assay (ELISA) and chemiluminescent immunoassay (CLIA).^7−9^ These two methods require the recognition of the primary antibody and secondary antibody during the immunoassay, which might take hours with redundant steps for washing and binding processes. Biosensors using colorimetric and electrochemical-based methods are also proposed.^10−15^ However, the materials for the experimental design include additional enzymes, nanozymes, and substrates to amplify the signals.

Additionally, plasmonic nanoparticles used in localized surface plasmon resonance (LSPR) offer alternatives with advantages such as simple processing, label-free detection, and rapid response times.^10,16^ Traditional LSPR techniques typically rely on peak shifts or aggregation-induced color changes as detection signals. However, these methods often require high analyte concentrations to generate a distinguishable signal, which can be insufficient for certain biomarker levels, as seen in sepsis diagnosis. For instance, in healthy individuals, plasma IL-6 levels are typically below 7 pg/mL.^5,17^ Furthermore, the complex nature of the blood plasma matrix adds challenges to the detection sensitivity and specificity.

To enhance the limit of detection (LOD) of LSPR-based methods, various analytical approaches are emerging to address the challenges posed by the low concentrations of certain biomarkers. Examples include ratiometric spectral analysis and digital spectral LSPR image analysis.^18−20^ Ratiometric analysis compares the absorbance intensities of monodispersed and aggregated plasmonic nanoparticles in UV–vis absorption spectra, typically at 550 and 650 nm, respectively. Digital spectral LSPR image analysis, on the other hand, assesses the brightness of individual AuNPs in scattering images across two adjacent wavelength regions centered around the LSPR peak. In a previous study, it was demonstrated that examining individual gold nanoparticles (AuNPs) achieved a three-order magnitude improvement in the LOD, reaching levels as low as 10 pg/mL. However, the system configuration—a complex dual-view imaging system combined with an ultrahigh-sensitivity monochrome scientific complementary metal–oxide–semiconductor (sCMOS) camera—poses challenges for practical applications due to its complexity and high cost. Additionally, the intricate nature of blood plasma and the low concentration of certain biomarkers present further challenges.

In this study, we propose a simpler, more cost-effective, and sensitive digital spectral LSPR imaging system. Rather than focusing on the peak of the scattering spectral profile of AuNPs, we analyze changes in the region near the full width at half-maximum (fwhm), where scattering intensity varies most significantly during surface binding events. By combining a metal halide lamp with multiple band-pass filters and a color camera, we capture each individual AuNP’s scattering intensities across two nonadjacent wavelength bands: one at 600 nm (R-band), near the fwhm, and the other at 550 nm (G-band), slightly shifted from the LSPR peak. The R-band is primarily responsible for signal response, while the G-band serves as a self-reference. To enhance system sensitivity and minimize noise, we further introduce spectral image contrast as a metric to evaluate each AuNP’s LSPR response.^21^ This approach enables precise, color-based measurement of LSPR responses, facilitating broader applications in biomolecule detection, such as monitoring IL-6 levels in blood plasma.

For IL-6 detection, AuNPs are modified with IL-6 monoclonal antibodies to capture IL-6 in samples. When IL-6 binds to the AuNP surface, different scattering intensities change within two wavelength bands close to the fwhm’s, which are recorded and analyzed. This platform demonstrates high sensitivity and precision in detecting IL-6 in complicated human blood plasma, achieving an LOD as low as 19.2 fg/mL and a 96% recovery rate within a 45-min detection time. These results align with those of commercial Multiplex Immunoassay Systems for IL-6 detection in human plasma samples. The sensing system thus holds promise for clinical applications, providing rapid and accurate IL-6 monitoring for sepsis patient care.

## Experimental Section

### Chemicals and Reagents

Poly(ethylene glycol) bis(amine) (PEG-(NH_2_)_2_, *M*_w_ 2,000), Tris-EDTA (TE) buffer (pH 8), trichloro(1*H*,1*H*,2*H*,2*H*-perfluorooctyl)silane (PTOCTS), and fetal bovine serum (FBS) were purchased from Sigma-Aldrich, Taiwan. Citrate-capped spherical gold nanoparticles (AuNPs) with a diameter of 80 ± 9 nm (Lot number: RXG0022, coefficient of variation = 10.9%) were purchased from nanoComposix, U.S. Recombinant human interleukin 6 (IL-6) was purchased from Elabscience (PKSH500018), and U.S. Mouse antihuman IL-6 monoclonal antibody (mAb_IL-6_) was purchased from PROSPEC (ant-109).

### Modification of AuNPs

Surface modification of mAb_IL-6_ on AuNPs (mAb_IL-6_@AuNPs) is based on a direct adsorption method. In brief, 500 μL of 80 nm bare AuNPs with an initial concentration of 1 × 10^10^ particles/mL was centrifuged at 5,000 rpm for 7 min at 4 °C, followed by replacing the supernatant with an equal volume of mAb_IL-6_ (50 μg/mL, dissolved in TE buffer) to redisperse the AuNPs pellet. Then, the solution was left to stand at 4 °C for 15 min, and 25 μL of PEG-(NH_2_)_2_ (1 mg/mL, dissolved in TE buffer) was further added to block the rest of the AuNPs surfaces (final weight ratio (w/w) of mAb_IL-6_ to PEG-(NH_2_)_2_ is 1:1). The mixture was incubated at 4 °C for 12 h to complete the modification. After that, the AuNPs were washed with TE buffer to remove unbound mAb_IL-6_ and PEG-(NH_2_)_2_. Finally, the pellet was resuspended with 30 μL TE buffer and stored at 4 °C as the stock solution for subsequent experiments.

### Process of IL-6 Detection

For standard curve establishment, deionized water (DI water) and stock FBS are spiked with various IL-6 concentrations. During detection, mAb_IL-6_@AuNPs are mixed with samples to reach a final concentration of 3.6 × 10^8^ particles/mL in the solution. Subsequently, the mixture is shaken at room temperature at 700 rpm for 30 min to allow the mAb-antigen capturing reaction to complete. The solution is then introduced into a microfluidic chip using a syringe pump set at a flow rate of 5 μL/min. The microfluidic chip is made according to the procedures from our previous research.^20^ In this step, the scattering light from AuNPs is collected by a color sCMOS camera (PCO. edge 4.2), the exposure time of the camera is 10 ms, with a frame rate set as 1 fps. A total data point of 15,000 light spots, the scattering of AuNPs, is recorded in each detection and analyzed by the MATLAB program.

### Pretreatment of Human Plasma Samples

The usage and research ethics of human plasma samples were reviewed by the Institutional Review Board for Biomedical Science Research, Academia Sinica, and approved with IRB project ID: AS-IRB-BM-24049. Human plasma samples were obtained from Boca Biolistics, and the blood cells were removed in advance. Before receiving the samples, all personal information and identification were delinked from the donor. Human plasma samples are first 10-fold diluted and centrifugally filtered through a filter membrane (Amicon Ultra-4 centrifugal filter, MWCO: 30 kDa, Merck) under 5,500 rpm for 10 min at 4 °C. Finally, the filtrate was collected for further analysis.

## Results and Discussion

### Dual-Segment Bandpass Wavelength Generated by Chromatic Digital Nanoplasmon-Metry (cDiNM)

**Figure 1:**
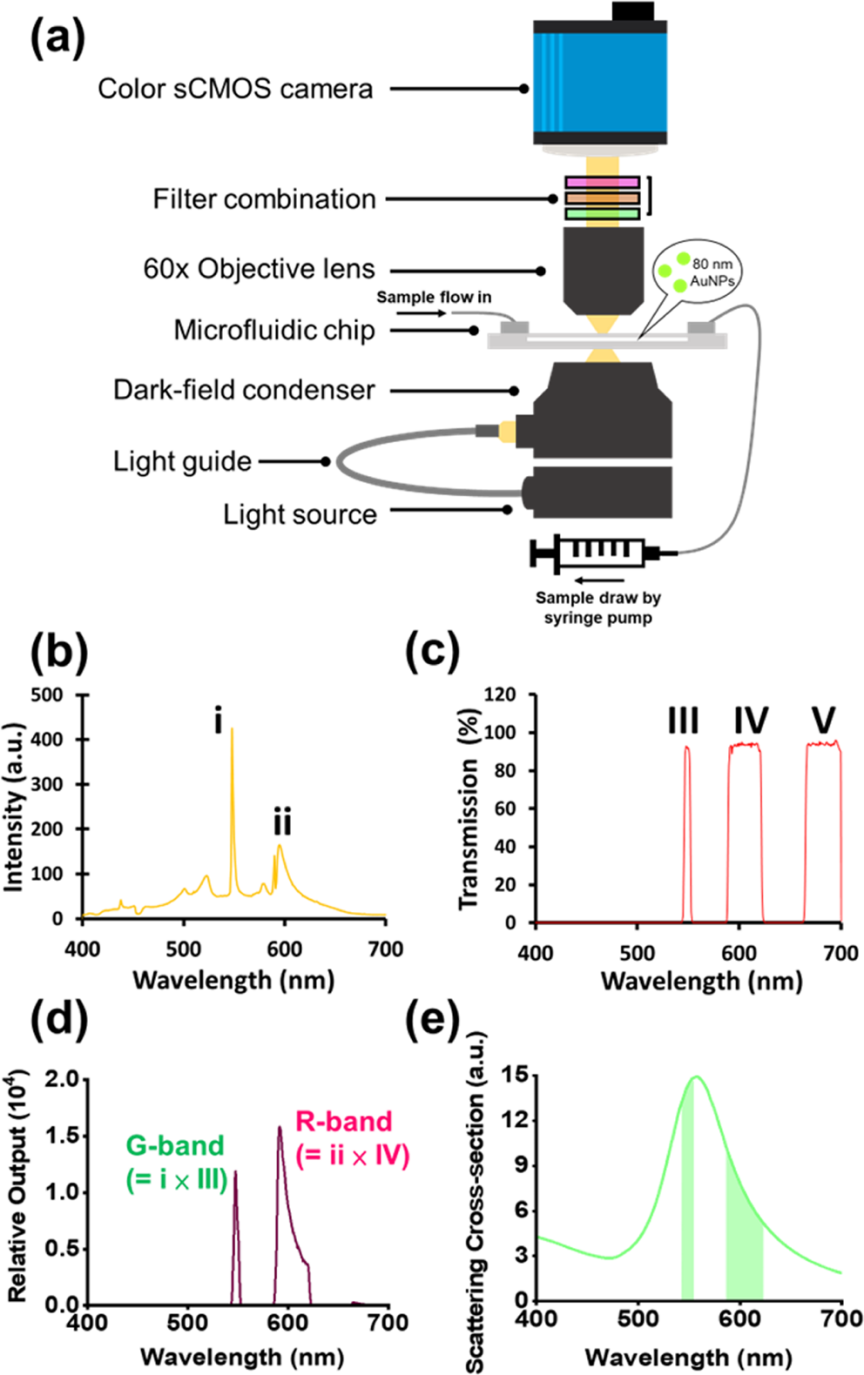
Setup of the cDiNM system. (a) Gold nanoparticles (80 nm) mixed with samples continuously flow through a microfluidic chip. The scattering light from the nanoparticles is excited by a dark-field condenser under the microfluidic chip, then emitted through the filter combination and recorded by the color sCMOS camera. (b) Spectrum of the metal halide lamp, with i and ii indicating the two strongest intensity peaks in the spectrum. (c) Overall transparency spectrum after the light penetrates through the filter combination. (d) The transmission spectra of the light passed through the filter combination. (e) Scattering spectrum from 80 nm gold nanoparticles. The two green areas under the crest indicate the overlapping of penetrating spectra from (d) with the scattering spectrum.

The cDiNM is composed of three major functional parts: a metal halide light source, a microfluidic system, and a color imaging system (Figure 1a). The sensing principle of the cDiNM is based on a dual-segment sensing approach, which compares the difference in signal intensity between two bandpass peaks. To produce suitable peaks for cDiNM, a filter combination composed of a multiband bandpass filter (MBPF, transmission wavelength: 417–433.5 nm, 466.5–492.5 nm, 525.5–552 nm, 590–622 nm, and 666.5–721 nm), a 525 nm long-pass filter (525-LPF), and a 532 nm notch filter (532-NF) is used. The MBPF permits transmission across five wavelength bands (Figure S1a, labeled I, II, III, IV, V). The addition of the 525-LPF and 532-NF further narrows these bands, reducing them to three (Figure S1d, labeled III, IV, and V).

Notably, the two strongest peaks of the mercury metal halide lamp (Figure 1b, labeled i and ii) align precisely with two of the wavelength bands defined by the filter combination (III and IV in Figures 1c and S1d). Beyond 650 nm, the luminance of the metal halide lamp decreases significantly, rendering the intensity negligible. This effectively reduces the three output spectral bands to two, as shown in Figure 1d. As a result, when light from the mercury metal halide lamp passes through the filters, only two nonadjacent wavelength bands remain (defined as the G-band and R-band in Figure 1d), which correspond to the green and red channels of the color sCMOS camera.

In cDiNM, the intensity of the dual-segment bandpass wavelength is measured within the overlapping spectrum produced by the metal halide lamp, the scattering light of AuNPs, and the transparency of the filter combination. For 80 nm AuNPs, the scattering spectrum peaks at 560 nm.^22^ Here, we compute the scattering light of 80 nm AuNPs based on Mie theory.^23^ As shown in Figure 1e, one segment (R-band) spans 590–622 nm, covering the fwhm of the longer wavelengths, while the other segment (G-band) is in the 545–552 nm range, slightly blue-shifted from the LSPR peak. The emitted scattering light is then collected by a 60× objective lens, passed through the filter combination, and recorded by a color sCMOS camera. Notably, scattering images of AuNPs passing through the G-band and R-band are captured simultaneously, being separately recorded in the green and red channels of the color sCMOS camera, respectively.

Compared to the previous dual-view setup that used a dichroic mirror to split two spectral images onto a monochrome sCMOS camera,^20^ this configuration integrates a mercury metal halide lamp—which provides distinct spectral peaks—with a multiband-pass filter and a color camera, making the system simpler and more cost-effective.

### Digital Spectral Chromatic Image Analysis of Self-referenced LSPR Changes from Individual AuNPs

In comparison to SPR, LSPR exhibits a shorter evanescent plasmon field at the surface of the AuNPs. This confined electromagnetic decay length enhances sensitivity to target molecules binding at the surface while reducing susceptibility to environmental interferences, such as variations in the buffer refractive index. Consequently, LSPR offers improved sensitivity and reduced matrix effects during detection. However, achieving optimal sensitivity requires careful consideration of the compatibility between the LSPR properties of the AuNPs and the experimental setup. According to Mie scattering theory,^23^ the LSPR peak wavelength and scattering intensity are strongly influenced by the diameter of the AuNPs, where AuNPs with smaller diameters have scattering intensities too weak to be reliably captured by the camera, and their LSPR peaks are misaligned with the G- and R-bands selected by the multiple band-pass filter set. Larger AuNPs, while producing sufficiently strong scattering intensities, may also exhibit LSPR peaks that are misaligned with the G- and R-bands. To address these constraints, 80 nm AuNPs were chosen for demonstration in this work.

**Figure 2:**
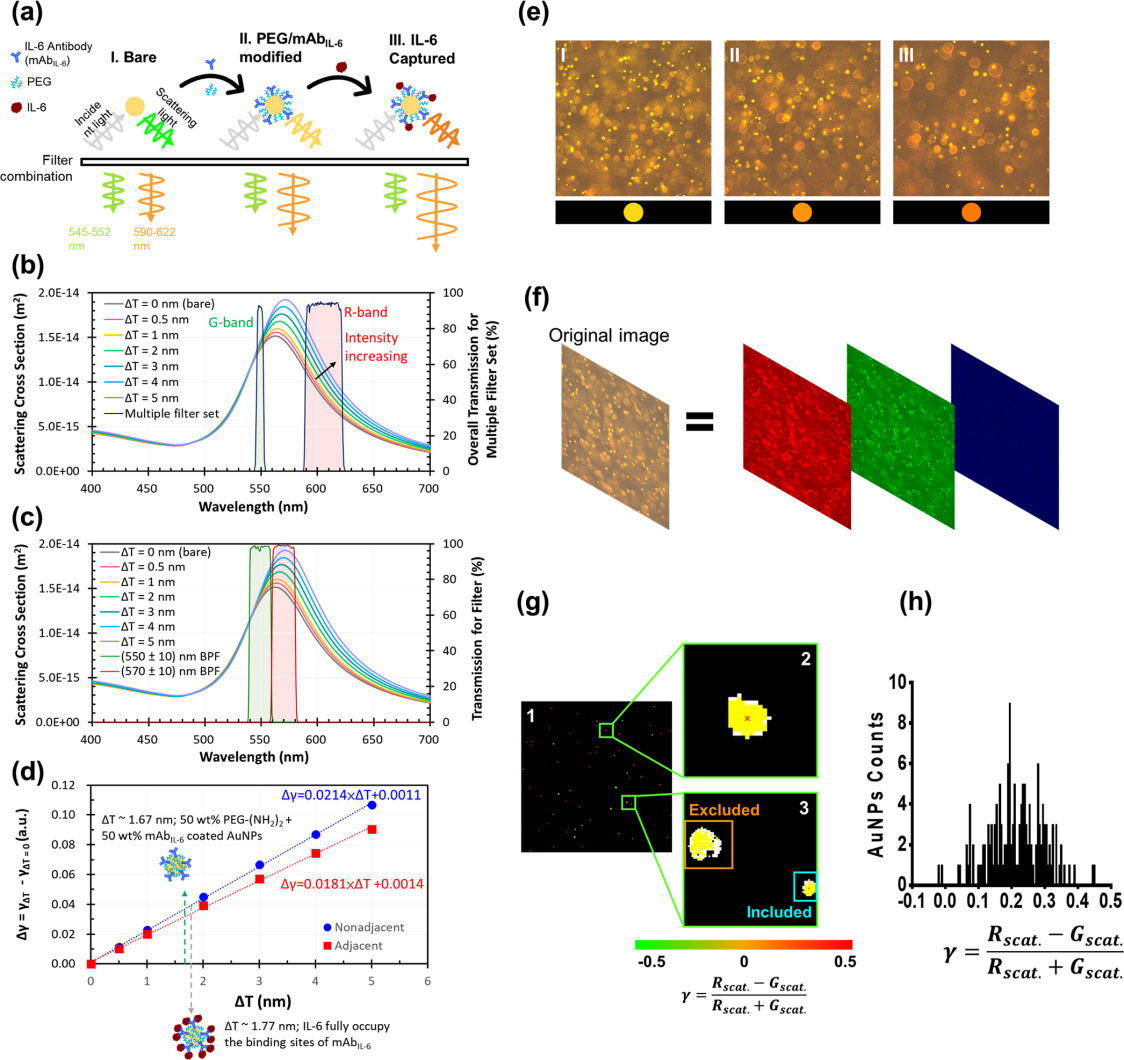
Work principium of the cDiNM system. (a) Modification of AuNPs and their changes in the scattering light intensity. Computed scattering cross sections of 80 nm-AuNPs with diverse protein covering thickness (Δ*T*) by Mie theory with (b) two nonadjacent transmission band produced by the filter combination and color sCMOS camera and (c) two adjacent transmission band produced by two band-pass filters and dual-view system. (d) Calculated surface sensitivity of (b,c). (e) Images of AuNPs’ color from sCMOS camera. (f) Image analysis of the red-shifted scattering light intensity change in the red and green channel. (g) Determine the spectral chromatic image contrast γ from each AuNPs. 1: Indicating the light spot by MATLAB, 2: The selected spot is marked with a red cross, 3: The oversized spot is excluded, while the normal-sized spot is included. (h) Statistics of γ values and the corresponding AuNPs count in the image.

In this study, IL-6 detection is achieved using 80 nm AuNPs modified with mAb_IL-6_ and PEG (Figure 2a). The modification is validated through UV–visible absorption spectra, dynamic light scattering (DLS) including hydrodynamic diameter and the polydispersity index (PDI), and X-ray photoelectron spectroscopy (XPS) (Figures S2 and S3 and Table S1). After the scattered light passes through the filter combination, dual-segment bandpass wavelengths emerge in the ranges of 545–552 nm and 590–622 nm, defined as the G band and R band, respectively (Figure 2b). Figure 2b also illustrates the calculated scattering cross-section of 80 nm AuNPs with various binding protein thicknesses (Δ*T*) modeled via Mie theory.^23^ As molecules bind to the AuNP surface (with increasing Δ*T*), the scattering of red light from LSPR shows a notably greater enhancement compared with green light scattering.

Here, we introduce the concept of spectral chromatic image contrast, referred to as the gamma (γ) value, to facilitate the evaluation of LSPR changes in a single AuNP. This is achieved by comparing the scattering intensities passing through the G-band and R-band, using the following equation:
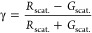
1where *R*_scat._ and *G*_scat_ denote the scattering intensities in the R-band and G-band, respectively. These represent the detected intensities from light spots in the R and G channels of the color sCMOS camera in the images.

Figure 2c presents scattering cross sections identical to those in Figure 2b, but with an adjacent G-band (540–560 nm) and R-band (560–580 nm) centered at the LSPR peak of 560 nm, as defined in a previous dual-view system.^20^ According to eq 1, the surface sensitivity *S*_s_ of the two systems was calculated and compared, as shown in Figure 2d, where *S*_s_ is defined by the following equation:
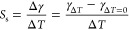
2

The results demonstrate that the nonadjacent system exhibits approximately 1.2 times greater sensitivity compared with the adjacent system. Specifically, when bare AuNPs are coated sequentially with PEG-(NH_2_)_2_ and mAb_IL-6_ (layer 1), followed by IL-6 (layer 2), the molecular thicknesses (Δ*T*) are approximately 1.67 nm for layer 1 and 1.77 nm for the combined layer 1 and layer 2, as calculated in Table S2.^24,25^ This configuration yields an 18% signal enhancement relative to that of the adjacent system. The improvement is attributed to the selection of spectral bands, where the narrower G-band in the nonadjacent system undergoes a smaller intensity change. This reduces the offset in the R-band’s increase, as described by eq 1.

Meanwhile, the broader R-band, covering the full width at half-maximum (fwhm), provides greater intensity changes, as seen in eq 1. Here, *G*_scat_ serves as a self-reference for the signal *R*_scat._ used in eq 1. Additionally, (*R*_scat._ + *G*_scat._) not only normalizes the spectral chromatic image contrast but also effectively reduces the noise. This outcome demonstrates a simpler, more cost-effective, and, most importantly, more sensitive setup.

A color sCMOS camera equipped with cDiNM aims to capture the scattering light changes from the AuNPs. Figure 2e shows the actual view from the camera during scattering light capture; each light spot in the image indicates one AuNP. The orange color is caused by AuNPs scattering light passing through the filter combination. Due to molecules binding, the red shift of the scattered light can be observed as the color of a spot turns from light orange to deep orange. Subsequently, the captured color image is further processed and analyzed using MATLAB to remove the background and extract the AuNPs scattering only. In brief, the original color image is divided into three individual color channels: red (R), green (G), and blue (B) (Figure 2f). Since the filter combination completely restricts the blue-light spectral wavelength, no clear light spot emerges in the B channel. Then, the spectral chromatic image contrast value, the gamma (γ) value, from each local brightest light spot, which indicates the individual AuNPs, is calculated according to eq 1. The red cross indicates the center of the tracked AuNPs. Given that color image analysis individually computes the AuNPs may involve aggregated AuNPs that contribute outlier γ values, we set a pixel threshold to exclude the oversized spot, while the normal-sized spot is included (Figure 2g). Finally, the statistics of frequent γ values and the corresponding amount of AuNPs count in the image are presented (Figure 2h).

### The Variation in AuNPs Count as a Signal for IL-6 Detection

**Figure 3:**
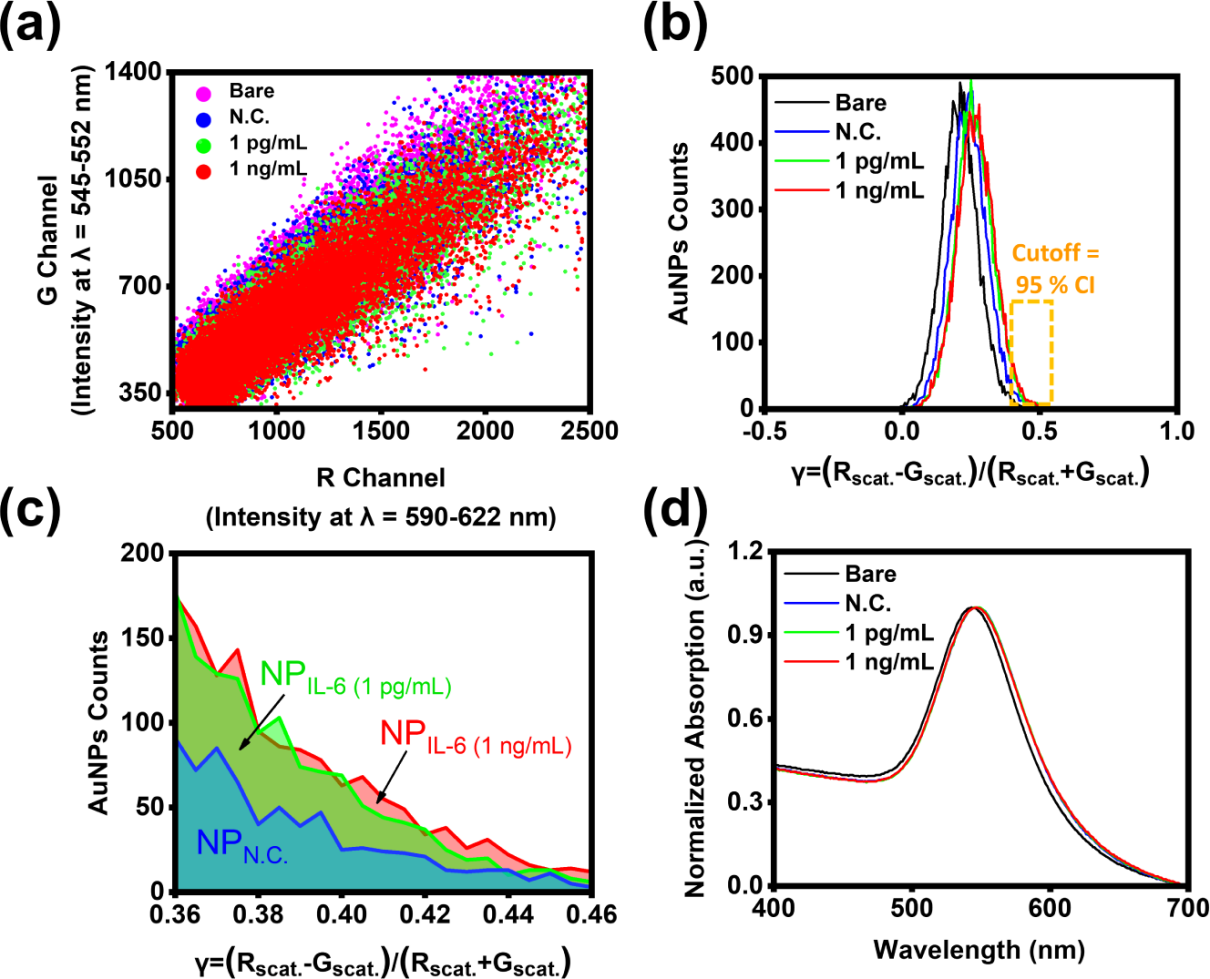
Calculation of the difference of AuNPs count number between negative control and IL-6 added group. (a) Distribution of *R*_scat._ and *G*_scat._ in the R and G channels that measured from 15,000 light spots. (b) Distribution of γ value from the calculations of the 15,000 light spots. (c) The difference of AuNPs count in the upper 95% confidence interval (CI) region. (d) The absorption wavelength from negative control and IL-6 added groups.

In this study, each AuNP continuously flowing through the microfluidic chip is captured by a color sCMOS camera to record the scattering light intensity *R*_scat._ and *G*_scat_. Within each data set, a total of 15,000 light spots are recorded to have sufficient statistical significance. Figure 3a shows the distribution of recorded scattering light intensity *R*_scat._ and *G*_scat._ of AuNPs. Each data point represents one light spot in the scattering images. It should be noted that the increasing PDI (as shown in Table S1) from bare AuNPs to mAb_IL-6_@AuNPs suggests that some degree of particle agglomeration occurs during the modification process. To mitigate its impact on IL-6 detection, the mAb_IL-6_@AuNPs are used as the negative control (N.C.) group instead of bare AuNPs.

Accompanied by the mAb_IL-6_ modification and IL-6 binding, the distribution of data point intensity is shifting from the G channel toward the R channel, followed by calculating the spectral chromatic image contrast to convert the intensity disparity from the G and the R channel into γ values, and the statistic of γ value from each light spot results in a final Gaussian distribution (Figure 3b). The distribution of the γ value shows a distinct right-sided shift compared to bare AuNPs due to the red-shifted scattering light increasing the intensity in *R*_scat._, while *G*_scat._ is reduced. The phenomenon can be explained by the red-shifted scattering light wavelength increasing the R band intensity and decreasing the G band intensity. Finally, the change in intensity from the R and G bands will separately contribute to the overall intensity and be reflected as *R*_scat._ and *G*_scat._ in the R and G channels.

Although recording extra light spots is beneficial for increasing cDiNM sensitivity in detecting the slight change of *R*_scat._ and *G*_scat._, the balance between spots capturing time and sensitivity has been examined in advance. Moreover, when N.C. mixes with IL-6, the capture of IL-6 onto the AuNPs surface further enhances the intensity on *R*_scat._, which brings about a slight shift of the distribution. This result coincides with the former computed red shift scale of AuNPs in Figure 2b,c.

The disparity of red-shifted γ value distribution from IL-6 added groups in Figure 3b provides cDiNM with the crucial signal for IL-6 detection. By setting a cutoff that falls at the 95% confidence interval (CI) in N.C.,^20^ the difference in the number of AuNPs count rate (ΔNP%) in the upper 95% CI region between IL-6 added groups and N.C. is calculated with the following formula:
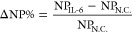
3where the NP_N.C._ and NP_IL-6_ represent the number of AuNPs count in the upper 95% CI region in N.C. and IL-6 added groups, respectively (Figure 3c). The elevated ΔNP% in Figure 3c indicates not only the enhanced red-shifted scattering light but also serves as the indicator to reflect an increase in IL-6 concentration in the sample.

Notably, due to the overlapping signals of N.C. and IL-6-spiked samples, distinguishing their overall responses using traditional analogue methods, such as averaging, is challenging. However, digital analysis enables the individual examination and extraction of relatively sparse signals (less than 5%) that are obscured by the average (more than 95%). To ensure statistically significant results, a large number of detections is crucial. In this work, a total of 15,000 detections (light spots) is achieved by analyzing the scattering images of flowing AuNPs. While this method may require a longer detection time (∼15 min) to collect a sufficient number of AuNPs compared to other approaches, such as FET-based sensors (detection time ∼4–5 min),^26^ it offers significant advantages in terms of simpler and more cost-effective sensor fabrication compared to both FET-based sensors and digital microwell array chips in biomolecule or pathogen detection.^27−29^

Digital analysis enables the detection of red shifts induced by IL-6 binding, even at low concentrations, using cDiNM. In contrast, traditional UV–vis spectroscopy, which measures the average light absorption, fails to reveal noticeable wavelength shifts between the N.C. and IL-6-spiked groups (Figure 3d). This result highlights the advantages of digital analysis in detecting ultralow concentrations.

### Sensitivity of cDiNM in IL-6 Detection

**Figure 4:**
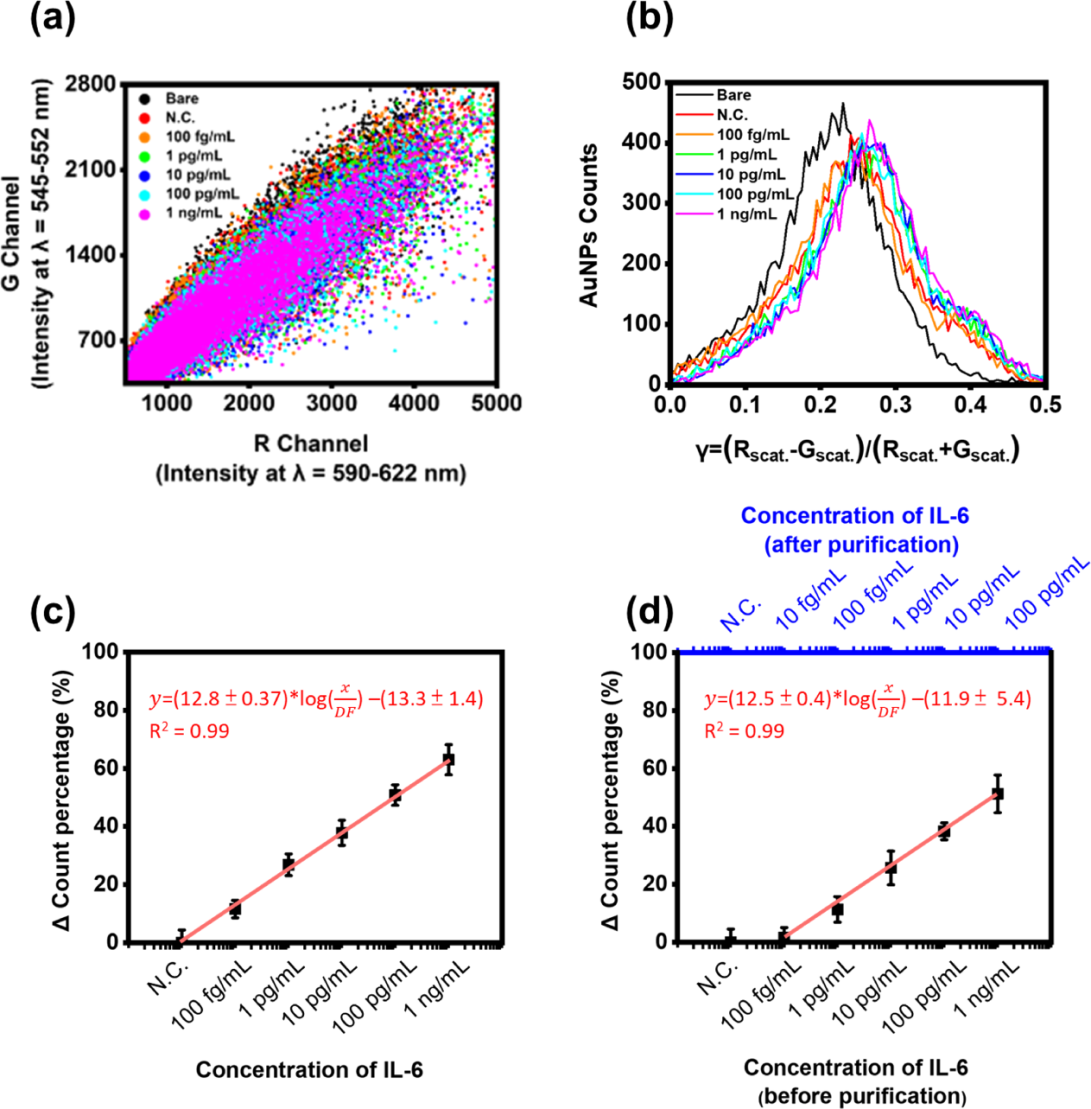
Validation of the sensitivity of cDiNM on IL-6 detection. (a) Shift of intensity between the G band and the R band under serial concentrations of IL-6. (b) Distribution of AuNPs counts and the corresponding γ value calculated from the intensity scattering plot of (a). (c,d) Standard curve of cDiNM detecting IL-6 in DI water and FBS, respectively (*n* = 6). The linear fitting between AuNPs count rate and IL-6 concentrations is demonstrated in red line. DF is the diluted factor, which is 1 and 10 in (c) and (d), respectively.

In healthy populations, the IL-6 concentration in blood is maintained at a lower level, less than 10 pg/mL. As a monitoring platform, the sensitivity of cDiNM to detecting IL-6 is evaluated. DI water was chosen as a simple environment, which was spiked with different concentrations of IL-6 to obtain the range that falls from 100 fg/mL to 1 ng/mL. The chosen concentration range includes IL-6 levels from individuals to patients with severe sepsis. As IL-6 concentration increases, scattering light from AuNPs changes from light orange to deep orange (Figure S4). However, the color change was negligible when the images were individually analyzed as the R and G channels. The cDiNM digitally converts the scattering light intensity from each light spot in the R and G channels. The change in intensity due to the LSPR red shift caused by IL-6 binding demonstrated its potential (Figure 4a). The contrast difference between the two channels is then converted into a γ value, which shifts toward the right as IL-6 concentration increases (Figure 4b). The AuNP count in the upper 95% CI region of the N.C. group is used as a cutoff with other IL-6 concentration groups to obtain the ΔNP%. Finally, the linear correlation between the ΔNP% and IL-6 concentration groups is derived from the data (Figure 4c).

The standard curves demonstrated a high linearity with *R*^2^ = 0.99. The limit of detection (LOD) and limit of quantification (LOQ) were 2.14 and 12.72 fg/mL, respectively, which were calculated according to eqs 4 and 5.^30,31^
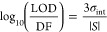
4
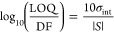
5

where DF is the diluted factor, σ_int_ is the standard deviation of the *y*-intercept, and *S* is the slope.

Human plasma contains proteins, lipids, and salts,^32^ which may cause serious matrix effects on the detection system. To further evaluate the cDiNM capability under a complex environment, FBS is applied to replace DI water to imitate a human plasma sample. The mAb_IL-6_ modified AuNPs remain stable in FBS during the detection process (Figure S5). When stock FBS flows through the microfluidic system, the impurities in the solution emit blue scattering light under the dark-field illumination system interfering with the sCMOS camera on *R*_scat._ and *G*_scat._ recording (Figure S6). Therefore, before measurement, each group spiked with IL-6 was 10-fold diluted (DF = 10), followed by centrifugation to remove uncorrelated impurities and reduce the blue color background. Although the dilution step reduces cDiNM sensitivity to IL-6, the resulting standard curve shows comparable linearity with the DI water condition, with an LOD and LOQ of approximately 19.2 and 87.8 fg/mL, which still covers the IL-6 concentration among healthy population and sepsis patients.

### IL-6 Monitoring in Human Blood Plasma

Clinically, monitoring IL-6 concentration in patients’ blood relies on ELISA and CLIA. This research proposes that cDiNM provides a time-saving, cost-effective, and label-free sensing approach as an alternative choice. As mentioned, the range of cDiNM on IL-6 detection has been investigated to fall in the concentration of 100 fg/mL to 1 ng/mL, whereas in healthy individuals, the concentration of IL-6 is lower than 10 pg/mL.

**Figure 5:**
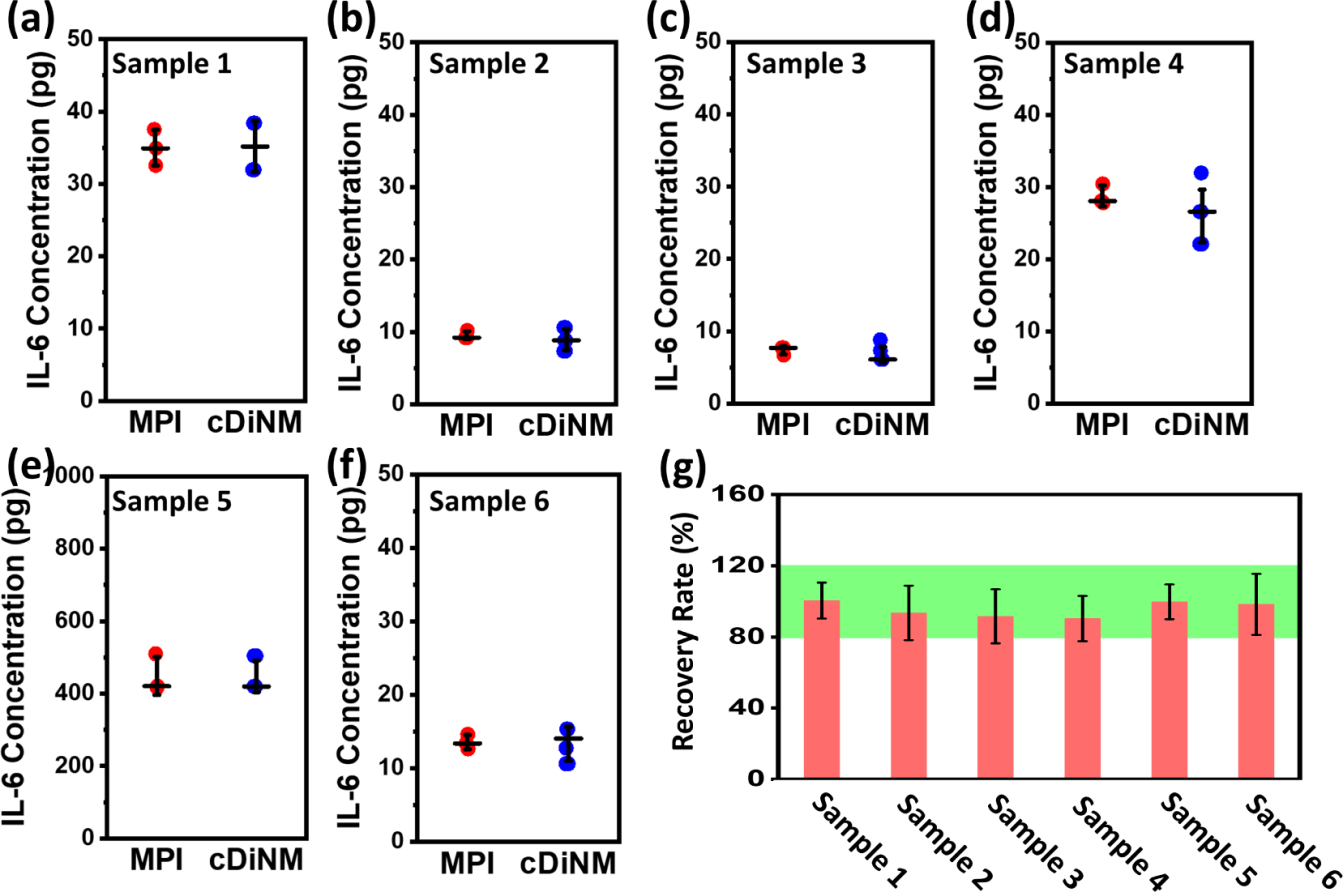
(a–f) IL-6 monitoring results in real human plasma. Samples 1–6 come from six respective donors. The replications for MPI are three repeats, while cDiNM is six repeats. (g) The recovery rates of samples 1 to 6. The green band indicates the acceptable ranges of 80–120%.

In total, six human plasma samples are used as real samples to assess the accuracy of cDiNM. The multiplex immunoassay (MPI), a widely used commercial method for determining IL-6 concentration, served as the benchmark for comparison. As shown in Figure 5a–f, the results of cDiNM show high consistency with the MPI. Further, the average recovery rate of IL-6 detected by cDiNM is 96% falling within the acceptable range between 80 and 120% (Figure 5g and Table S3), indicating the precision of cDiNM even in the complicated blood plasma, further presenting the potential for clinical monitoring of biomarkers in patients.

The enzyme-linked immunosorbent assay (ELISA) is widely regarded as the gold standard for protein detection. However, its complex and time-consuming procedures can limit its utility, particularly in cases where rapid clinical deterioration necessitates immediate monitoring. High-precision, rapid-response detection of critical biomarkers is essential for timely clinical intervention, potentially increasing patient survival rates. In recent years, various nanosensor-based detection tools have been developed to address these challenges. Table S4 summarizes several IL-6 detection methods, detailing their specifications, including limits of detection (LOD), sample volume requirements, and detection times.^10,15,33−39^ Among these, label-free methods such as electrochemistry (EC) and surface plasmon resonance (SPR) offer faster and simpler workflows, but their sensitivity can be affected by matrix effects. For the chemiluminescence and photoelectrochemical immunoassays, although they provide faster detection compared to ELISA, the substrates can be costly, and the complexity of the required equipment may pose other limitations. Conversely, cDiNM exhibits high sensitivity for IL-6 detection. Nonetheless, achieving consistent modification of gold nanoparticles (AuNPs) with antibodies while maintaining good particle monodispersity plays a key role in the detection.

Overall, each detection method has distinct advantages and limitations. The approach presented in this study demonstrates exceptional performance for IL-6 detection in complex environments, as made evident by the comprehensive evaluation provided.

## Conclusion

As a cytokine to mediate physiological functions, IL-6 not only plays an important role in immune responses to regulate leukocyte activities and tissue inflammation but is also a potential factor in energy metabolism control. These diverse effects of IL-6 make it a significant biomarker in the clinical field. Therefore, we aimed to develop a rapid, highly sensitive, and straightforward method for the identification of the IL-6 level in the complex environment of human blood plasma, providing a novel option for monitoring changes in IL-6 concentration in clinical practice.

In this study, we used cDiNM to detect changes in the LSPR of AuNPs, induced by biomolecules attaching to their surfaces. The LSPR of AuNPs enables label-free detection. The cDiNM setup incorporates a filter combination and a color sCMOS camera that selectively allows light within two nonadjacent, specific wavelength bands to pass through. One band is centered within the fwhm range where the LSPR response is strongest, while the other band is narrowed and blue-shifted from the peak to a region with a minor intensity change. This configuration allows every AuNPs’ spectral chromatic image contrast to be a self-referenced subtractive analysis LSPR that enhances its sensitivity and simplifies the signal acquisition.

Subsequently, cDiNM digitally analyzes the spectral chromatic image contrast across numerous individual AuNPs, significantly enhancing the signal output even at low IL-6 levels in complicated human blood plasma. Overall, cDiNM effectively detects IL-6 within 45 min, requiring only 0.5 mL of a 10-fold diluted, label-free sample, with an LOD and LOQ of less than 19.2 and 87.8 fg/mL, respectively, and a recovery rate of 96%. It demonstrates reliable performance in measuring IL-6 concentrations in plasma samples from both healthy individuals and those at risk of sepsis. This platform shows potential as an efficient tool for clinical biomarker monitoring.

## Supporting Information Available

Spectrum generated from the filter combination, UV–vis absorption spectra, dynamic light scattering (DLS), zeta potentials (*V*_ζ_), XPS spectra, scattering images, stability, matrix influence test results, method comparison, and recovery rate (PDF)
