# Supplementary material for: Self-referenced Digital Spectral Chromatic Local Surface Plasmon Resonance in Ultrasensitive Severe Sepsis Interleukin-6 Detection
Source: ACS Sens. 2025 Feb 5;10(2):1178–86. doi: 10.1021/acssensors.4c03067 (PMC11877628; doi:10.1021/acssensors.4c03067)
Supplement: Supplementary file 1 — se4c03067_si_001.pdf [file se4c03067_si_001.pdf]

Supporting Information

Self-Referenced Digital Spectral Chromatic Local Surface Plasmon Resonance in Ultrasensitive Severe Sepsis Interleukin-6 Detection.

Ting-Wei Chang,[a,b,c, ‡] Ting-Hao Chuang,[a,d, ‡] Sheng-Hann Wang,[a, ‡] Wing Kiu Yeung,[d, *] and Pei-Kuen Wei[a, *]

*Corresponding author:

W. K. Yeung: vicki.yeung@mail.ntut.edu.tw; P. K. Wei: pkwei@gate.sinica.edu.tw

[a]Research Center for Applied Sciences, Academia Sinica, Taipei 115201, Taiwan.

[b]Nano Science and Technology Program, Taiwan International Graduate Program, Academia Sinica, Taipei 11529, Taiwan.

[c]Department of Engineering and System Science, National Tsing Hua University, Hsinchu 300, Taiwan.

[d]Department of Materials and Mineral Resources Engineering, National Taipei University of Technology, Taipei 10608, Taiwan.

1. Spectrum generated from the filter combination

A mercury metal halide lamp is applied as light source for cDiNM. However, the characteristic of line spectrum from the lamp makes it difficult to illustrate the spectrum characteristic of individual filter. Therefore, a halogen light source (Hong-Ming Technology, Taiwan) with a continuous wavelength range is chosen as light source here for demonstration. Figure S1b, S1c, and S1e separately show the spectrum of each filter, while S1d and S1f show the alteration of spectrum at different stage of the incident light passing through the filters.

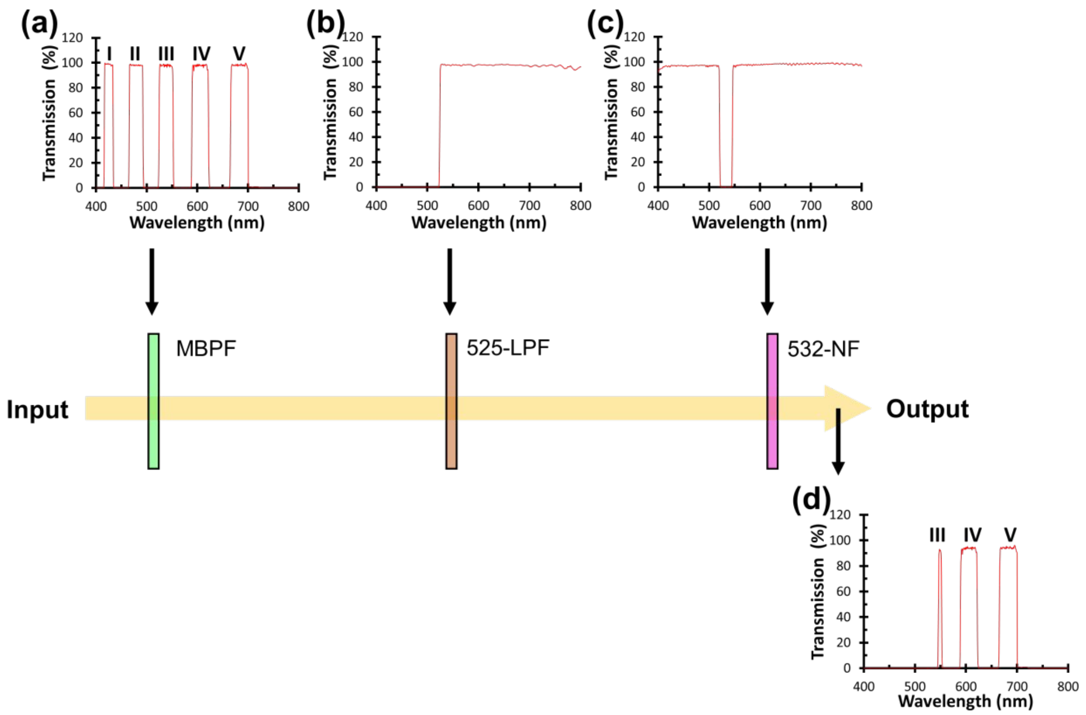

Figure S1: Transmission spectra of (a) multiple band pass filter (MBPF), (b) 525 nm long-pass filter, (c) 532 nm notch filter(532-NF), and (d) the overall filter combination.

2. UV-Vis absorption spectra, Dynamic light scattering (DLS), and Zeta potentials (V_ζ)

The alteration of UV-Vis absorption spectra, hydrodynamic diameter (D_h) and surface electrical potential (V_ζ) of AuNPs is analyzed via BioTek Micropalte Reader, NanoBrook 90Plus particle size analyzer (Brookhaven Instruments, USA). As figure S2a-b depicts, AuNPs modified with PEG-$(NH_2)_2$ (PEG@AuNPs) shows a slightly red-shift in spectra and increased D_h in compared with bare AuNPs. When AuNPs modified with mAb_{IL-6} and PEG-$(NH_2)_2$ (mAb_{IL-6}@AuNPs), an obvious 5-nm peak-shift and increase of D_h around 133.3 nm occurs. This may come from mAb binds to AuNPs that contributes a large molecular weight layer on the surface.

In liquid phase, due to the negatively-charged carboxylate ion groups from dissociation of citrate acid, the citrate-capped bare AuNPs expresses negative charge V_ζ on its surface (Fig S2b). When modified with PEG-$(NH_2)_2$ (PEG@AuNPs), an increased V_ζ comes from positive amine groups from the attached PEG-$(NH_2)_2$.[1] In comparison with PEG@AuNPs, the V_ζ from mAb_{IL-6}@AuNPs doesn't show obvious change. The reason may come from immunoglobulin G (IgG) that we applied in this research has the isoelectric point (pI) at around 6.4 to 7.6,[2] which expresses weak negative to neutral charge.

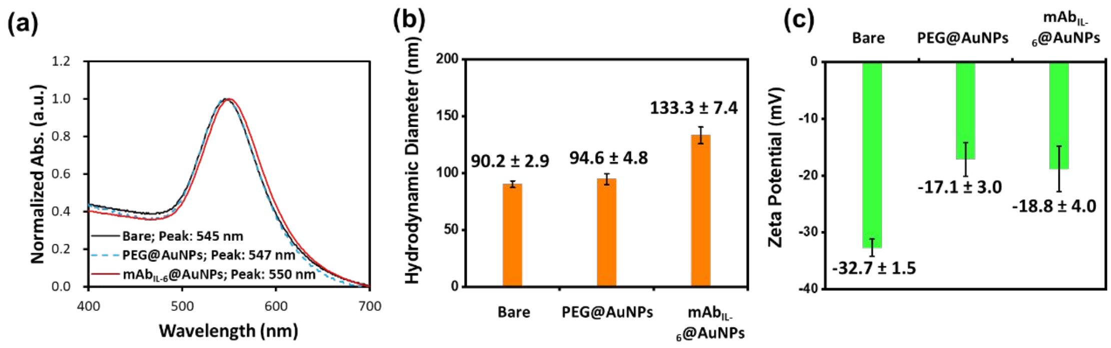

Figure S2: (a) The UV-vis absorption spectra, (b) hydrodynamic diameter, and (c) zeta potential (V_ζ) of bare, PEG, and mAb_{IL-6} modified AuNPs (n=3).

Table S1. Polydispersity index of nanoparticles from each individual measurement

	Polydispersity index (PDI)	Mean of PDI	Std. error of PDI
Bare-1	0.063	0.058	0.005
Bare-2	0.053		
Bare-3	0.057		
PEG@AuNPs-1	0.079	0.095	0.014
PEG@AuNPs-2	0.105		
PEG@AuNPs-3	0.100		
mAb_{IL-6}@AuNPs-1	0.138	0.124	0.013
mAb_{IL-6}@AuNPs-2	0.112		
mAb_{IL-6}@AuNPs-3	0.121		

3. X-ray photoelectron spectroscopy (XPS)

The XPS spectra were measured via PHI5000 VersaProbe Scanning XPS System (ULVAC-PHI Inc., Japan). The microfocused Al K_α X-rays (25 W, 100 μm) was chosen as radiation source, with a take-off angle of the photoelectron fixed at 45°. The measurement was conduct in an ultrahigh vacuum chamber (1×10^{-5} Pa) for spectroscopic analysis. As supplementary figure S3a shows, a slightly increased of the binding energy of gold from IL-6 mAb modified group depicts the present of extra layer on AuNPs surface in compared with bare. Moreover, a distinct enhanced binding energy of S2p profile in IL-6 mAb modified group shows the existence of sulfur (Figure S3b). The element may come from amino acid such as cysteine that contains sulfur, which plays a critical role to form disulfide bonds in protein structure.[3] The result indicates the successful modification of mAb on AuNPs surface.

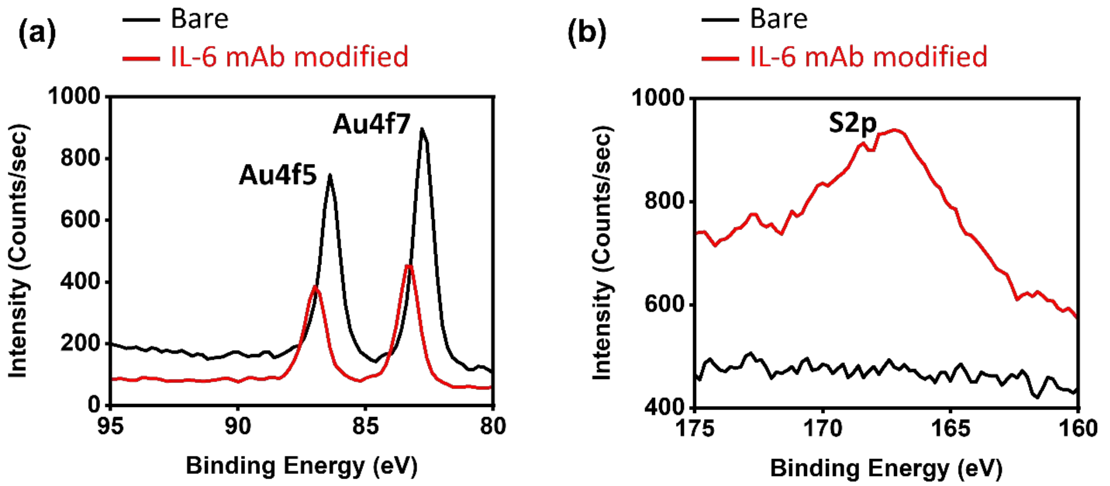

Figure S3: (a) Au 4f XPS spectra from bare and IL-6 mAb modified AuNPs. (b) S 2p XPS spectra from bare and IL-6 mAb modified AuNPs.

4. Surface thickness estimation of the mAB_{IL-6}, PEG-$(NH_2)_2$, and IL-6 coated AuNPs

Table S2. Surface thickness estimation of the mAB_{IL-6}, PEG-$(NH_2)_2$, and IL-6 coated AuNPs.

	Component			Weight Ratio	Molar Ratio (M/M)	Weighted - Averaged Thickness (ΔT)
	Name	Molecular Weight (Mw)	Diameter[4]			
**Layer 1**	mAb_{IL-6}	~150 kDa	7 nm	50 wt%	$\frac{\frac{50\,wt\%}{150}}{\frac{50\,wt\%}{150}+\frac{50\,wt\%}{2}} = \frac{1}{76}$	$7\,(nm) \times \frac{1}{76} + 1.6\,($ $\times \frac{75}{76} \cong 1.67\,nm$
	PEG-$(NH_2)_2$	2 kDa	1.6 nm	50 wt%	$\frac{\frac{50\,wt\%}{2}}{\frac{50\,wt\%}{150}+\frac{50\,wt\%1}{2}} = \frac{75}{76}$	
**Layer 2**	IL-6	21 - 26 kDa[5]	3.6 – 3.9 nm (average = 3.75 nm)	-	$\frac{1}{76} \times 2^{*}$	$3.75\,(nm) \times \frac{2}{76} \cong 0.098\,nm$ ~ 0.1 nm

*Every mAB_{IL-6} provides 2 binding sites to IL-6

5. Change of AuNPs scattering light

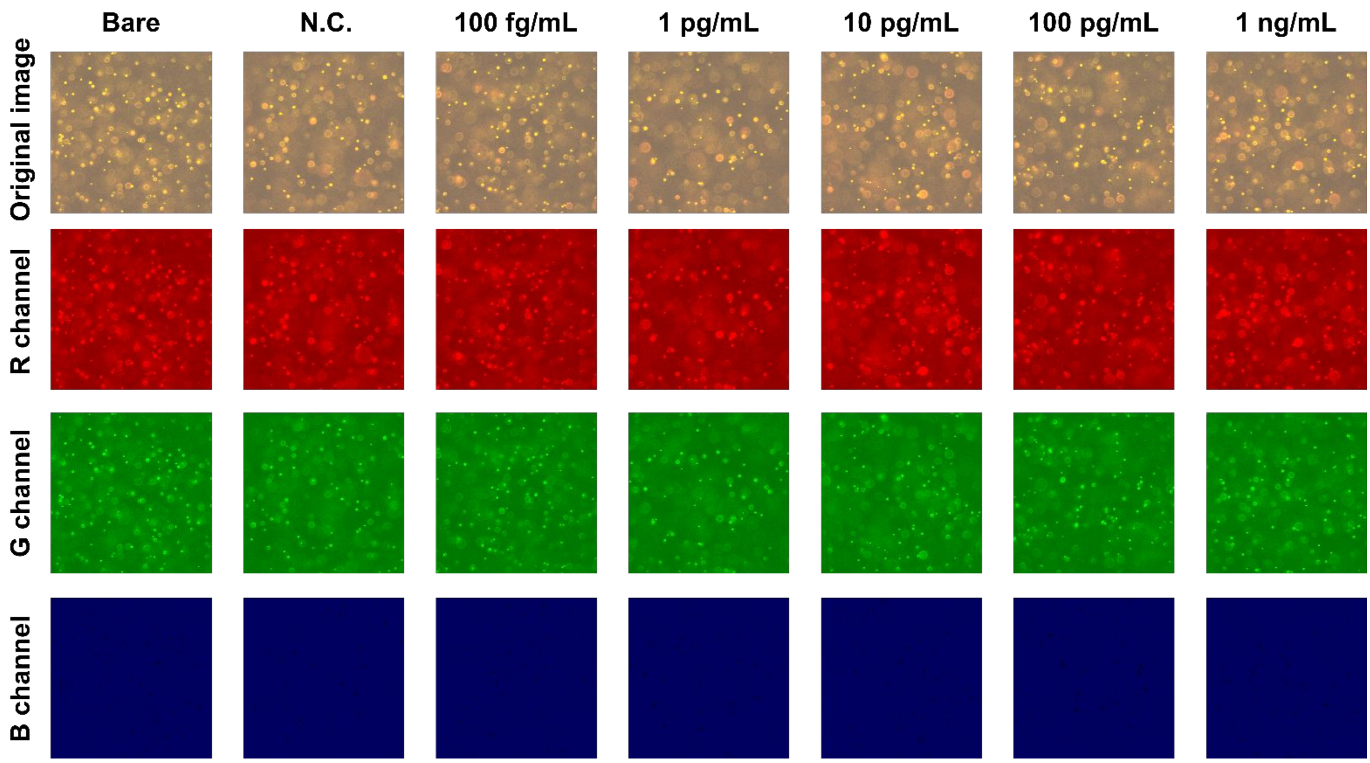

Figure S4: The change of AuNPs scattering light in serial concentrations of IL-6 when observed under original color image and separated R, G, and B channel.

6. Stability of IL-6 mAb modified AuNPs in FBS

The stability of IL-6 mAb modified AuNPs is examined by separately placing same concentration of AuNPs in 1X TE buffer and stock FBS, then 700 rpm shaken for 30 minutes to mimic the matrix environment during IL-6 detection. After 30 minutes, the solution was directly measured with UV–Vis absorption spectrometer (Biotek Synergy 2). The result shows that no obvious red shift of the wavelength that caused by the aggregation or molecules binding onto AuNPs surface is observed.

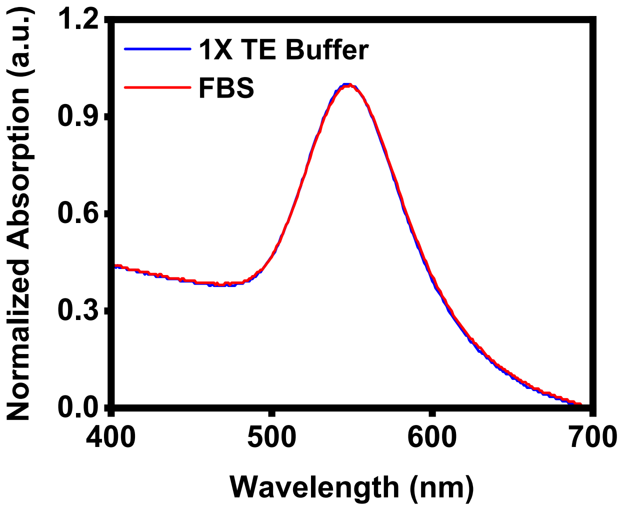

Figure S5: The normalized absorption spectrum of IL-6 mAb modified AuNPs that separately present in 1X TE buffer and stock FBS.

7. The matrix effect of scattering light from FBS

To demonstrate the interference of FBS scattering light to cDiNM, the filter combination was removed here to allow color sCMOS camera capturing initial color images. When DI water flow through the microfluidic chip, no scattering light was observed under the dark-field illumination system. However, stock FBS which contains various impurities demonstrates distinct blue background with blue white scattering light spots from large debris. The color comes from the effect of dielectric in FBS.[6] These debris would contribute inescapable scattering light intensity to $R_{scat.}$ and $G_{scat.}$, which could interfere cDiNM on γ value analysis. Therefore, the purification step is required to significantly reduce the effect of dielectric debris to cDiNM.

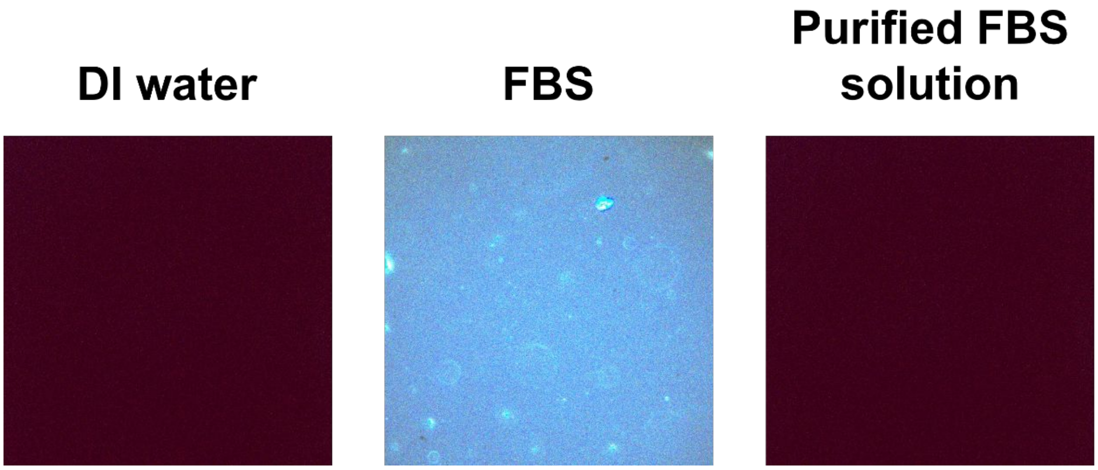

Figure S6: Observation of scattering light from different solutions under the dark-field illumination system.

8. Recovery rate

Table S3. Recovery rate of cDiNM detects IL-6 in human plasma.

Repeat #	Sample 1	Sample 2	Sample 3	Sample 4	Sample 5	Sample 6
1	109.7%	111.2%	82.7%	92.4%	93.5%	113.2%
2	109.7%	92.5%	82.7%	92.4%	93.5%	113.2%
3	109.7%	92.5%	82.7%	76.9%	112.4%	78.3%
4	91.3%	77.0%	119.5%	76.9%	93.5%	78.3%
5	91.3%	77.0%	82.7%	111.1%	93.5%	113.2%
6	91.3%	111.2%	99.4%	92.4%	112.4%	94.1%

9. Method comparison of IL-6 detection

Table S4. Method comparison of IL-6 detection

	Method	LOD	Matrix	Sample volume	Detection time	Ref.
1	Self-referenced digital spectral image contrast of LSPR	19.2 fg/mL	10 % Human blood plasma	0.5 mL (0.05 mL ,before diluted)	45 min.	This work
2	Enzyme-linked immunosorbent assay (ELISA)	92 fg/mL	Human blood plasma	0.1 mL	> 4 hours	[7]
3	Chemiluminescence immunoassay (CLIA)	0.5 pg/mL	Human serum	0.1 mL	75 min.	[8]
4	Surface plasmon resonance (SPR)	1.3 ng/mL	Conditioned medium	0.06 mL	20 min.	[9]
5	Photoelectrochemical immunoassay	0.38 pg/mL	Buffer	0.01 mL	60 min.	[10]
6	AuNPs aggregation-induced color changes	1.95 µg/mL	Mixed protein solution	0.3 mL	5 min.	[11]
7	Microfluidic electrochemical magnetoimmunosensor	0.42 pg/mL	Human serum	0.04 mL	Overnight	[12]
8	Electrochemistry	10 pg/mL	10 % human serum	-	30 min.	[13]
9	Electrochemical surface plasmon resonance (EC-SPR)	10 pg/mL	Cell culture condition medium	0.15 mL	60 min.	[14]
10	Single-walled carbon nanotube (SWCNT)-based fluorescence and absorbance spectrometry	25 pg/mL	Human serum	-	30 min.	[15]

Reference

(1) Fukushima, O.; Tsutsumi, Y.; Hanawa, T. Mechanism of Electrodeposition Process of Poly(Ethylene Glycol) Diamine to Titanium Surface. *MATERIALS TRANSACTIONS* **2020**, *61* (7), 1346-1354. DOI: 10.2320/matertrans.MT-M2020111.

(2) Danielsson, Å.; Ljunglöf, A.; Lindblom, H. One-step purification of monoclonal IgG antibodies from mouse ascites: An evaluation of different adsorption techniques using high performance liquid chromatography. *Journal of Immunological Methods* **1988**, *115* (1), 79-88. DOI: https://doi.org/10.1016/0022-1759(88)90312-2.

(3) Bulaj, G. Formation of disulfide bonds in proteins and peptides. *Biotechnology*

Advances **2005**, *23* (1), 87-92. DOI: https://doi.org/10.1016/j.biotechadv.2004.09.002.
(4) Erickson, H. P. Size and Shape of Protein Molecules at the Nanometer Level Determined by Sedimentation, Gel Filtration, and Electron Microscopy. *Biological Procedures Online* **2009**, *11* (1), 32. DOI: 10.1007/s12575-009-9008-x.
(5) Tanaka, T.; Narazaki, M.; Kishimoto, T. IL-6 in inflammation, immunity, and disease. *Cold Spring Harbor perspectives in biology* **2014**, *6* (10), a016295. DOI: https://10.1101/cshperspect.a016295.
(6) Wang, S.-H.; Lee, C.-W.; Chiou, A.; Wei, P.-K. Size-dependent endocytosis of gold nanoparticles studied by three-dimensional mapping of plasmonic scattering images. *Journal of Nanobiotechnology* **2010**, *8* (1), 33. DOI: 10.1186/1477-3155-8-33.
(7) Martin, K.; Viera, K.; Petr, C.; Marie, N.; Eva, T. Simultaneous analysis of cytokines and co-stimulatory molecules concentrations by ELISA technique andof probabilities of measurable concentrations of interleukins IL-2, IL-4, IL-5, IL-6, CXCL8 (IL-8), IL-10, IL-13 occurring in plasma of healthy blood donors. *Mediators of Inflammation* **2006**, *2006* (1), 065237. DOI: https://doi.org/10.1155/MI/2006/65237.
(8) Luo, L.; Zhang, Z.; Hou, L.; Wang, J.; Tian, W. The study of a chemiluminescence immunoassay using the peroxyoxalate chemiluminescent reaction and its application. *Talanta* **2007**, *72* (4), 1293-1297. DOI: https://doi.org/10.1016/j.talanta.2007.01.030.
(9) Chou, T.-H.; Chuang, C.-Y.; Wu, C.-M. Quantification of Interleukin-6 in cell culture medium using surface plasmon resonance biosensors. *Cytokine* **2010**, *51* (1), 107-111. DOI: https://doi.org/10.1016/j.cyto.2010.04.004.
(10) Fan, G.-C.; Ren, X.-L.; Zhu, C.; Zhang, J.-R.; Zhu, J.-J. A new signal amplification strategy of photoelectrochemical immunoassay for highly sensitive interleukin-6 detection based on TiO2/CdS/CdSe dual co-sensitized structure. *Biosensors and Bioelectronics* **2014**, *59*, 45-53. DOI: https://doi.org/10.1016/j.bios.2014.03.011.
(11) Giorgi-Coll, S.; Marín, M. J.; Sule, O.; Hutchinson, P. J.; Carpenter, K. L. H. Aptamer-modified gold nanoparticles for rapid aggregation-based detection of inflammation: an optical assay for interleukin-6. *Microchimica Acta* **2019**, *187* (1), 13. DOI: 10.1007/s00604-019-3975-7.
(12) Zhang, C.; Shi, D.; Li, X.; Yuan, J. Microfluidic electrochemical magnetoimmunosensor for ultrasensitive detection of interleukin-6 based on hybrid of AuNPs and graphene. *Talanta* **2022**, *240*, 123173. DOI: https://doi.org/10.1016/j.talanta.2021.123173.
(13) Sánchez-Salcedo, R.; Miranda-Castro, R.; de-los-Santos-Álvarez, N.; Lobo-Castañón, M. J.; Corrigan, D. K. Comparing nanobody and aptamer-based capacitive sensing for detection of interleukin-6 (IL-6) at physiologically relevant levels. *Analytical and Bioanalytical Chemistry* **2023**, *415* (29), 7035-7045. DOI: 10.1007/s00216-023-04973-4.

(14) Lo, S.-C.; Wang, S.-H.; Chang, T.-W.; Lee, K.-L.; Chern, R.-L.; Wei, P.-K. Dual Gold-Nanoslit Electrodes for Ultrasensitive Detection of Antigen–Antibody Reactions in Electrochemical Surface Plasmon Resonance. *ACS Sensors* **2022**, *7* (9), 2597-2605. DOI: 10.1021/acssensors.2c00850.
(15) Gaikwad, P.; Rahman, N.; Parikh, R.; Crespo, J.; Cohen, Z.; Williams, R. M. Optical Nanosensor Passivation Enables Highly Sensitive Detection of the Inflammatory Cytokine Interleukin-6. *ACS Applied Materials & Interfaces* **2024**, *16* (21), 27102-27113. DOI: 10.1021/acsami.4c02711.
